# Infection with bovine leukemia virus belonging to group A or B-1 contributes more strongly to the development of enzootic bovine leukosis in young cattle than the presence of bovine lymphocyte antigen*-DRB3* susceptibility alleles

**DOI:** 10.1007/s00705-024-06102-7

**Published:** 2024-08-01

**Authors:** Yuki Fujii, Masaki Maezawa, Masataka Akagami, Junko Kawakami, Yuri Fujimoto, Hisashi Inokuma

**Affiliations:** 1 Livestock Division, Ibaraki Prefecture Government, Mito, Ibaraki 310-8555 Japan; 2 Laboratory of OSG Veterinary Science for Global Disease Management, Graduate School of Agricultural and Life Sciences, The University of Tokyo, Bunkyo-ku, Tokyo, 113-8657 Japan; 3 Ibaraki Prefectural Kensei Livestock Hygiene Service Center, Chikusei, Ibaraki 300-4516 Japan; 4 Laboratory of Farm Animal Medicine, Graduate School of Agricultural and Life Sciences, The University of Tokyo, Bunkyo-ku, Tokyo, 113-8657 Japan

## Abstract

In this study, we compared the effects of different bovine leukemia virus (BLV) strains and bovine lymphocyte antigen (*BoLA*)*-DRB3* alleles in cattle with enzootic bovine leukosis (EBL) aged either <3 years or ≥3 years. The frequency of infection with BLV belonging to group A or B-1 in cattle aged <3 years with EBL was significantly higher than that in cattle aged ≥3 years, regardless of which *BoLA-DRB3* allele was present. This suggests that infection with group A or B-1 BLV contributes more strongly to the development of EBL in young cattle than the presence of early-EBL-onset susceptibility *BoLA-DRB3* alleles.

**Supplementary Information:**

The online version contains supplementary material available at 10.1007/s00705-024-06102-7.

Enzootic bovine leukosis (EBL) is caused by bovine leukemia virus (BLV) [1]. More than 95% of BLV infections in cattle remain subclinical, but some animals develop persistent lymphocytosis, with fewer than 5% developing tumors [2]. Almost all cases of EBL involve cattle aged ≥3 years due to the lengthy latency period of BLV [3]. However, several cases of EBL in cattle aged <3 years have also been reported [4–6]. Dairy cattle typically have a calf for the first time when they are 2 years old and begin to produce milk. Because these young cows are still growing, mature cows that have had two or more deliveries typically produce more milk than those nursing their first calf [7]. Once beef cattle reach market weight at 2 to 3 years of age, they are slaughtered for meat production. Products from cattle with EBL are discarded for food safety reasons, and thus EBL in young cattle can cause extensive economic losses.

Both viral and host factors are involved in the development of EBL in young cattle [8, 9]. BLV strains can be divided into five groups (A, B-1, B-2, C, and other) based on their genome sequence, members of group A have been shown to replicate faster than other strains [10]. Moreover, 83.3% of BLV isolates from cattle aged <3 years with EBL belong to group A or B-1, while 48.0% of BLV isolates from cattle aged ≥3 years with EBL belong to group B-2 [8]. The major histocompatibility complex in cattle, referred to as "bovine lymphocyte antigen" (BoLA), is associated with the susceptibility to developing bovine lymphoma in the Holstein-Frisian (HF) and Japanese Black (JB) breeds [11, 12]. The frequency of detection of the BoLA-DRB3*15:01 allele in cattle aged <3 years EBL with has been found to be 37.3% (48.0% and 29.4% in HF and JB, respectively) and to be significantly higher than that (11.6%) in cattle over 3 years old. Moreover, in HF cattle, the frequency of detection of the BoLA-DRB3*16:01 allele in cattle aged <3 years with EBL has been reported to be 20.0%, which is significantly higher than that in cattle over 3 years old (1.3%). Therefore, those alleles have been identified as early-EBL-onset susceptibility alleles in HF and JB cattle [10]. However, it is unclear which factor is the most important for predicting economic losses due to EBL. In the present study, we compared the effects of different BLV strains and *BoLA-DRB3* susceptibility alleles in cattle aged <3 years with EBL and those aged ≥3 years to assess their relative contributions to the development of EBL in young cattle.

Peripheral blood, lymph nodes, and solid tumors from several organs were collected from 76 cattle with EBL (46 HF, 20 JB, and 10 crossbreeds of HF and JB; F1) aged <3 years and 56 cattle with EBL (32 HF and 24 JB) aged ≥3 years. The presence of B-cell lymphoma was confirmed by pathological examination and/or a B-cell clonality test [13]. BLV copy numbers in all cattle were over 2,000 copies/50 ng DNA, which was used as the diagnostic criterion for association of BLV with tumor development in a previous study [14]. Genomic DNA was extracted from peripheral blood and tissues using a QIAamp DNA Mini Kit (QIAGEN, GmbH, Hilden, Germany) and stored at -30°C.

BLV strains were identified as described previously [8]. Briefly, BLV-specific PCR was performed using two primer pairs (BLV 1-17 + BLV 4565-4586 and BLV 4416-4436 + BLV 8703-8720) [8]. DNA libraries were prepared using a QIAGEN QIAseq FX DNA Library Kit (QIAGEN) and sequenced on an Illumina iSeq system using 2 × 150 bp paired-end reads. Mapping of quality-filtered reads against a reference BLV genome sequence (accession no. EF600696.1) was performed for determination of the complete genome sequences of BLV isolates using CLC Genomics Workbench v. 20.0 (CLC bio, Aarhus, Denmark) (CLC). Phylogenetic analysis was performed using complete genome sequences of BLV isolates from animals with EBL and sequences obtained from the GenBank database (accession numbers LC733243, LC733245, LC733248, LC733314 to LC733317, LC733320, LC733321, LC733323, LC733327, LC733360, AP0180012, AP0180016, and AP0180018).

*BoLA-DRB3* genotyping was performed as described previously [9]. Tailed PCR was performed using genomic DNA and primer pairs (NGS-DRB3FRW + NGS-DRB3RE) [9]. DNA libraries were prepared using a Nextera XT DNA Library Prep Kit (Illumina) and sequenced on an Illumina MiSeq system using 2 × 300 bp paired-end reads. *BoLA-DRB3* sequences were downloaded from the IPD-MHC database (https://www.ebi.ac.uk/ipd/mhc/group/BoLA/), and mapping of quality-filtered reads against *BoLA-DRB3* sequences was performed using CLC. Homozygotes and heterozygotes were distinguished based on a previous report [9]. In the present study, the BoLA-DRB3*15:01 allele was considered an early-EBL-onset susceptibility allele in the HF, JB, and F1 breeds [9]. In addition, the BoLA-DRB3*16:01 allele was defined as an early-EBL-onset susceptibility allele in the HF breed [9].

Tables 1, 2 and 3 show the association of EBL with different BLV strains, different *BoLA-DRB3* genotypes, and combinations of different BLV strains and *BoLA-DRB3* genotypes, respectively. In phylogenetic analysis based on complete BLV genome sequences, 58 out of 76 BLV isolates from cattle aged <3 years with EBL and 13 out of 56 BLV isolates from those aged ≥3 years belonged to group A or B-1 (Table 1). Nucleotide substitutions resulting in amino acid substitutions at Pro 158 (A and B-1: alanine) and Pro 233 of the Tax protein (A and B-1: leucine) were observed. In *BoLA-DRB3* genotyping, 80 of 152 alleles in cattle aged <3 years and 26 of 112 alleles of those aged ≥3 years were early-EBL-onset susceptibility alleles (Table 2). In cattle aged <3 years with EBL, the frequency of the combination of BLV group A or B-1 and an early-EBL-onset susceptibility allele was 39.5% (60 of 152) (Table 3). The frequency of the combination of BLV group A or B-1 and alleles other than early-EBL-onset susceptibility alleles was 36.8% (56 of 152) (Table 3). A chi-square test and analysis of adjusted residuals with EZR, a graphical user interface for R (The R Foundation for Statistical Computing, version 1.40) [15], revealed that the frequency of the combination of BLV group A or B-1 and an early-EBL-onset susceptibility allele and of BLV group A or B-1 and alleles other than early-EBL-onset susceptibility alleles were both significantly higher in cattle less than 3 years old than those in cattle aged ≥3 years (*P* < 0.001). Using adjusted residuals analysis, no significant difference between cattle with EBL aged <3 years and ≥3 years was observed in the frequency of the combination of BLV isolates not belonging to group A or B-1 and early-EBL-onset susceptibility alleles. Moreover, the frequency of the combination of BLV isolates not belonging to group A or B-1 and alleles other than early-EBL-onset susceptibility alleles in cattle aged <3 years with EBL was significantly lower than that in cattle aged ≥3 years with EBL (*P* < 0.001, adjusted residuals analysis).

**Table 1:** Classification of BLV strains based on BLV whole-genome sequencing

	Cattle under 3 years old with EBL (N = 76)	Cattle over 3 years old with EBL (N = 56)
Group A or B-1	58 (76.3%)	13 (23.2%)
Group A	31	5
Group B-1	27	8
Other groups*	18 (23.7%)	43 (76.8%)
Group B-2	10	28
Group C	1	0
Other	7	15

**Table 2:** *BoLA-DRB3* genotyping of cattle with EBL

Holstein-Friesian					Japanese Black and F1*				
*BoLA-DRB3* alleles	Cattle aged <3 years with EBL (N = 92)		Cattle aged ≥3 years with EBL (N = 64)		*BoLA-DRB3* alleles	years with EBL (N = 60)		years with EBL (N = 48)	
	n	%	n	%		n	%	n	%
Early-EBL-onset susceptibility alleles	56	60.9	18	28.1	Early-EBL-onset susceptibility alleles	24	40.0	8	16.7
*15:01*	33			14	*15:01*	24		8	
*16:01*	23			4					
Other alleles**	36	39.1	46	71.9	Other alleles**	36	60.0		40
*01:01*	3			8			*01:01*	2	
*05:03*	1			0			*02:01*	1	
*07:01*	0			2			*05:02*	1	
*08:01*	3			0			*05:03*	2	
*09:02*	0			1			*05:04*	1	
*10:01*	3			2			*07:01*	0	
*11:01*	9			16			*10:01*	1	
*12:01*	8			6			*11:01*	2	
*140:11*	5			4			*12:01*	1	
*27:03*	2			7			*140:11*	2	
*44:01*	2			0			*16:01*	19	
							*27:03*	4	

^*^F1: crossbreeds of Holstein-Frisian and Japanese Black

^**^Other alleles: Alleles other than early-EBL-onset susceptibility alleles

**Table 3:** Combination of different BLV strains and *BoLA-DRB3* genotypes

	Group A or B-1		Other strains*	
	Early-EBL-onset susceptibility alleles	Other alleles**	Early-EBL-onset susceptibility alleles	Other alleles**
Cattle under 3 years old with EBL (N = 152)	60 (39.5%)	56 (36.8%)	20 (13.2%)	16 (10.5%)
Cattle over 3 years old with EBL (N = 112)	11 (9.8%)	15 (13.4%)	15 (13.4%)	71 (63.4%)

^*^Other strains: BLV strains other than groups A and B-1

^**^Other alleles: other than early-EBL-onset susceptibility alleles

We analyzed BLV strains and *BoLA-DRB3* alleles in cattle aged <3 years and ≥3 years with EBL to determine which factors contribute more to the development of EBL in young cattle. As reported previously, BLV strains belonging to group A or B-1 and early-EBL-onset susceptibility alleles were mainly observed in cattle aged <3 years with EBL [8, 9]. These results suggest that infection with BLV of group A or B-1 and the presence of an early-EBL-onset susceptibility allele contributes to the development of EBL in young cattle. Moreover, the frequency of infection by BLV strains belonging to group A or B-1 in cattle aged <3 years with EBL was significantly higher than that in those aged ≥3 years, regardless of their *BoLA-DRB3* allele status, suggesting that the genotype of the virus contributes more to the development of EBL in young cattle than the presence of an early-EBL-onset susceptibility allele. Therefore, elimination of animals infected with group A and B-1 strains might be more effective than the exclusion of early-EBL-onset susceptibility *BoLA* alleles in preventing EBL in young cattle. However, because amino acid mutations within the gag and *env* genes can alter the binding affinity of individual *BoLA-DRB* alleles to the virus, further investigation of the relationship between BLV strains and BoLA-DRB3 alleles is needed.

In conclusion, the present study showed that infection by BLV strains belonging to group A and B-1 or the presence of early-EBL-onset susceptibility alleles may independently contribute to the development of EBL in cattle aged <3 years. Moreover, infection with group A and B-1 strains might contribute more to the onset of EBL in young cattle than the presence of early-EBL-onset susceptibility alleles. This study might contribute to the establishment of more-efficient measures to reduce economic losses caused by EBL.

### Supplementary Information

Below is the link to the electronic supplementary material.

Supplementary file1 (PDF 70 KB)

Supplementary file2 (PDF 87 KB)

## Data Availability

The datasets generated and/or analyzed in the current study are available from the corresponding author upon reasonable request.
