# Supplementary material for: Infection with bovine leukemia virus belonging to group A or B-1 contributes more strongly to the development of enzootic bovine leukosis in young cattle than the presence of bovine lymphocyte antigen-DRB3 susceptibility alleles
Source: Arch Virol. 2024 Aug 1;169(8):171. doi: 10.1007/s00705-024-06102-7 (PMC11294373; doi:10.1007/s00705-024-06102-7)
Supplement: Supplementary file 1 — Supplementary file1 (PDF 70 KB) [file 705_2024_6102_MOESM1_ESM.pdf]

Supplemental table S1. Results of classifying BLV strains and BoLA-DRB3 genotyping in EBL cattle aged <3 years

Cattle ID	Age (month)	Sex	Breed	Sample	Location	BLV proviral load (copies/50 ng DNA)	BLV Group	Accession No.	BoLA-DRB3
EBL1	7	F	JB	Solid tumor	Hokkaido	12,002	A	LC817102	*15:01/15:01*
EBL2	8	M	F1	Lymph node	Hokkaido	4,223	B-1	LC817103	*15:01/15:01*
EBL3	10	F	HF	Peripheral blood	Chiba	5,204	B-1	LC817104	*16:01/16:01*
EBL4	13	M	HF	Lymph node	Hokkaido	6,622	A	LC817105	*15:01/15:01*
EBL5	13	F	HF	Lymph node	Ibaraki	8,329	Other	LC817106	*01:01/08:01*
EBL6	13	F	HF	Peripheral blood	Ibaraki	4,747	Other	LC817107	*01:01/11:01*
EBL7	14	M	HF	Peripheral blood	Ibaraki	20,257	A	LC817108	*12:01/12:01*
EBL8	14	F	HF	Lymph node	Ibaraki	2,427	Other	LC817109	*11:01/44:01*
EBL9	15	M	F1	Solid tumor	Ibaraki	4,091	B-2	LC817110	*15:01/15:01*
EBL10	15	M	HF	Lymph node	Ibaraki	6,793	B-1	LC817111	*15:01/15:01*
EBL11	17	F	HF	Lymph node	Ibaraki	8,166	B-1	LC817112	*16:01/16:01*
EBL12	17	F	HF	Peripheral blood	Ibaraki	17,287	B-1	LC817113	*15:01/15:01*
EBL13	18	F	HF	Lymph node	Ibaraki	6,333	A	LC817114	*15:01/15:01*
EBL14	18	F	JB	Lymph node	Ibaraki	2,633	B-2	LC817115	*15:01/15:01*
EBL15	18	F	HF	Lymph node	Ibaraki	4,602	A	LC817116	*12:01/12:01*
EBL16	19	F	HF	Peripheral blood	Ibaraki	10,053	A	LC817117	*15:01/15:01*
EBL17	19	F	HF	Lymph node	Ibaraki	2,366	B-1	LC817118	*05:03/08:01*
EBL18	19	M	HF	Lymph node	Hokkaido	4,577	A	LC817119	*15:01/15:01*
EBL19	19	F	HF	Lymph node	Hokkaido	19,502	Other	LC817120	*15:01/15:01*
EBL20	19	F	HF	Lymph node	Ibaraki	4,922	A	LC817121	*11:01/11:01*
EBL21	20	F	HF	Lymph node	Ibaraki	6,595	A	LC817122	*12:01/12:01*
EBL22	20	F	JB	Lymph node	Ibaraki	13,322	A	LC817123	*15:01/15:01*
EBL23	20	F	JB	Lymph node	Ibaraki	11,718	B-1	LC817124	*16:01/16:01*
EBL24	20	M	JB	Solid tumor	Ibaraki	2,567	A	LC817125	*16:01/16:01*
EBL25	20	F	HF	Solid tumor	Ibaraki	7,438	B-1	LC817126	*140:11/140:11*
EBL26	20	F	HF	Peripheral blood	Ibaraki	8,235	A	LC817127	*16:01/16:01*
EBL27	21	F	HF	Lymph node	Ibaraki	2,937	A	LC817128	*16:01/16:01*
EBL28	21	M	HF	Lymph node	Ibaraki	4,747	B-1	LC817129	*15:01/15:01*
EBL29	21	M	HF	Lymph node	Ibaraki	16,759	B-1	LC817130	*11:01/15:01*
EBL30	21	F	HF	Lymph node	Ibaraki	4,500	B-1	LC817131	*15:01/27:03*
EBL31	21	F	JB	Lymph node	Ibaraki	16,321	A	LC817132	*01:01/15:01*
EBL32	21	M	JB	Lymph node	Ibaraki	2,969	B-1	LC817133	*15:01/140:11*
EBL33	21	M	F1	Lymph node	Ibaraki	3,529	B-2	LC817134	*15:01/16:01*
EBL34	22	M	F1	Lymph node	Ibaraki	8,311	Other	LC817135	*11:01/11:01*
EBL35	22	F	F1	Peripheral blood	Ibaraki	6,968	B-1	LC817136	*05:03/12:01*
EBL36	22	F	HF	Lymph node	Ibaraki	10,902	B-1	LC817137	*11:01/11:01*
EBL37	22	M	HF	Lymph node	Ibaraki	2,266	B-2	LC817138	*16:01/16:01*
EBL38	23	M	JB	Peripheral blood	Ibaraki	8,963	B-1	LC817139	*15:01/15:01*
EBL39	23	F	JB	Lymph node	Ibaraki	6,441	B-2	LC817140	*16:01/16:01*
EBL40	23	F	HF	Lymph node	Ibaraki	5,966	A	LC817141	*15:01/16:01*
EBL41	23	F	F1	Peripheral blood	Ibaraki	7,888	B-1	LC817142	*16:01/16:01*
EBL42	23	F	F1	Lymph node	Ibaraki	12,203	A	LC817143	*02:01/27:03*
EBL43	23	F	JB	Lymph node	Ibaraki	40,832	A	LC817144	*05:02/15:01*
EBL44	23	M	HF	Lymph node	Ibaraki	5,182	A	LC817145	*16:01/16:01*
EBL45	24	M	JB	Lymph node	Ibaraki	4,633	B-2	LC817146	*16:01/16:01*
EBL46	24	F	JB	Peripheral blood	Ibaraki	7,032	A	LC817147	*05:03/15:01*
EBL47	24	F	F1	Lymph node	Ibaraki	26,001	A	LC817148	*10:01/15:01*
EBL48	25	M	JB	Solid tumor	Ibaraki	8,882	A	LC817149	*01:01/27:03*
EBL49	25	F	JB	Peripheral blood	Ibaraki	9,920	A	LC817150	*16:01/16:01*
EBL50	25	M	JB	Lymph node	Ibaraki	9,225	A	LC817151	*140:11/27:03*
EBL51	25	M	HF	Lymph node	Ibaraki	26,091	A	LC817152	*16:01/16:01*
EBL52	25	M	HF	Lymph node	Ibaraki	4,361	A	LC817153	*15:01/15:01*
EBL53	26	F	HF	Lymph node	Ibaraki	9,832	A	LC817154	*16:01/16:01*
EBL54	27	F	F1	Solid tumor	Hokkaido	3,199	B-2	LC817155	*15:01/15:01*
EBL55	27	F	HF	Lymph node	Hokkaido	9,361	A	LC817156	*10:01/10:01*
EBL56	27	F	JB	Peripheral blood	Ibaraki	4,017	B-1	LC817157	*15:01/15:01*
EBL57	27	F	HF	Peripheral blood	Ibaraki	2,734	B-1	LC817158	*10:01/11:01*
EBL58	27	F	HF	Lymph node	Chiba	10,610	C	LC775095	*140:11/15:01*
EBL59	27	M	JB	Lymph node	Hokkaido	7,391	B-1	LC817159	*16:01/16:01*
EBL60	27	F	JB	Lymph node	Ibaraki	9,072	B-1	LC817160	*15:01/15:01*
EBL61	27	F	HF	Lymph node	Ibaraki	9,843	A	LC817161	*12:01/16:01*
EBL62	27	F	HF	Peripheral blood	Hokkaido	7,653	B-1	LC817162	*11:01/16:01*
EBL63	27	M	HF	Peripheral blood	Hokkaido	8,473	A	LC817163	*16:01/16:01*
EBL64	28	M	JB	Peripheral blood	Ibaraki	7,428	B-1	LC817164	*16:01/16:01*
EBL65	28	M	HF	Lymph node	Ibaraki	13,072	B-2	LC817165	*15:01/15:01*
EBL66	28	M	HF	Lymph node	Ibaraki	9,834	A	LC817166	*08:01/27:03*
EBL67	30	M	JB	Solid tumor	Ibaraki	5,320	B-1	LC817167	*16:01/16:01*
EBL68	30	M	HF	Lymph node	Ibaraki	4,600	Other	LC817168	*16:01/16:01*
EBL69	31	M	HF	Lymph node	Ibaraki	7,328	Other	LC817169	*15:01/15:01*
EBL70	31	M	HF	Lymph node	Ibaraki	8,093	B-1	LC817170	*15:01/15:01*
EBL71	31	M	HF	Peripheral blood	Ibaraki	18,203	B-1	LC817171	*01:01/140:11*
EBL72	32	M	HF	Lymph node	Ibaraki	2,093	B-2	LC817172	*140:11/44:01*
EBL73	33	F	F1	Peripheral blood	Ibaraki	2,272	B-1	LC817173	*05:04/27:03*
EBL74	34	F	HF	Peripheral blood	Ibaraki	2,542	A	LC817174	*12:01/15:01*
EBL75	35	M	HF	Lymph node	Ibaraki	7,493	B-1	LC817175	*15:01/15:01*
EBL76	35	M	HF	Lymph node	Ibaraki	3,299	B-2	LC817176	*15:01/15:01*

HF: Holstein-Frisian, JB: Japanese Black, F1: crossbreeds of HF and JB
