# Supplementary material for: Infection with bovine leukemia virus belonging to group A or B-1 contributes more strongly to the development of enzootic bovine leukosis in young cattle than the presence of bovine lymphocyte antigen-DRB3 susceptibility alleles
Source: Arch Virol. 2024 Aug 1;169(8):171. doi: 10.1007/s00705-024-06102-7 (PMC11294373; doi:10.1007/s00705-024-06102-7)
Supplement: Supplementary file 2 — Supplementary file2 (PDF 87 KB) [file 705_2024_6102_MOESM2_ESM.pdf]

Supplemental table S2. Results of classifying BLV strains and BoLA-DRB3 genotyping in EBL cattle aged ≥3 years

Cattle ID	Age (month)	Sex	Breed	Sample	Location	BLV proviral load (copies/10 ng DNA)	BLV Group	Accession No.	BoLA DRB3
EBL77	36	F	HF	Lymph node	Hokkaido	3,326	Other	LC817177	*11:01/27:03*
EBL78	37	F	HF	Peripheral blood	Ibaraki	12,400	B-2	LC817178	*09:02/140:11*
EBL79	39	F	HF	Solid tumor	Ibaraki	9,284	B-2	LC817179	*27:03/27:03*
EBL80	39	F	HF	Lymph node	Ibaraki	12,223	Other	LC817180	*01:01/11:01*
EBL81	40	F	HF	Lymph node	Ibaraki	8,439	Other	LC817181	*11:01/11:01*
EBL82	40	F	HF	Lymph node	Ibaraki	9,994	B-1	LC817182	*27:03/27:03*
EBL83	41	F	JB	Peripheral blood	Ibaraki	9,352	B-2	LC817183	*16:01/16:01*
EBL84	43	F	HF	Lymph node	Ibaraki	4,844	A	LC817184	*15:01/15:01*
EBL85	44	F	HF	Lymph node	Ibaraki	5,638	B-1	LC817185	*01:01/01:01*
EBL86	45	F	JB	Lymph node	Ibaraki	3,375	Other	LC817186	*16:01/16:01*
EBL87	47	F	JB	Lymph node	Ibaraki	7,864	B-2	LC817187	*10:01/15:01*
EBL88	48	F	HF	Lymph node	Ibaraki	7,553	B-2	LC817188	*01:01/11:01*
EBL89	48	F	JB	Solid tumor	Ibaraki	2,044	Other	LC817189	*27:03/27:03*
EBL90	48	F	JB	Peripheral blood	Ibaraki	23,905	Other	LC817190	*16:01/16:01*
EBL91	49	F	JB	Lymph node	Ibaraki	9,530	B-2	LC817191	*16:01/16:01*
EBL92	50	F	HF	Lymph node	Ibaraki	6,223	B-1	LC817192	*12:01/12:01*
EBL93	52	F	HF	Lymph node	Ibaraki	4,297	B-2	LC817193	*12:01/12:01*
EBL94	54	F	HF	Lymph node	Ibaraki	3,935	A	LC817194	*11:01/140:11*
EBL95	54	F	JB	Lymph node	Ibaraki	3,082	Other	LC817195	*02:01/02:01*
EBL96	55	F	JB	Lymph node	Ibaraki	5,592	B-2	LC817196	*01:01/15:01*
EBL97	57	F	HF	Lymph node	Ibaraki	6,660	Other	LC817197	*11:01/27:03*
EBL98	57	F	HF	Lymph node	Ibaraki	5,590	B-2	LC817198	*15:01/15:01*
EBL99	59	F	HF	Peripheral blood	Chiba	2,421	B-2	LC817199	*11:01/11:01*
EBL100	60	F	HF	Lymph node	Ibaraki	8,599	B-2	LC817200	*140:11/15:01*
EBL101	62	F	HF	Lymph node	Ibaraki	9,785	B-2	LC817201	*15:01/15:01*
EBL102	66	F	HF	Lymph node	Ibaraki	7,111	B-2	LC817202	*01:01/01:01*
EBL103	69	F	HF	Lymph node	Ibaraki	4,555	B-1	LC817203	*01:01/16:01*
EBL104	70	F	JB	Peripheral blood	Ibaraki	2,216	Other	LC817204	*01:01/02:01*
EBL105	71	F	HF	Peripheral blood	Ibaraki	3,723	B-2	LC817205	*11:01/12:01*
EBL106	77	F	JB	Lymph node	Ibaraki	2,960	B-1	LC817206	*15:01/15:01*
EBL107	81	F	HF	Lymph node	Ibaraki	15,529	Other	LC817207	*07:01/07:01*
EBL108	83	F	JB	Lymph node	Ibaraki	4,722	B-2	LC817208	*10:01/10:01*
EBL109	83	F	HF	Lymph node	Ibaraki	4,002	A	LC817209	*11:01/12:01*
EBL110	84	F	HF	Lymph node	Ibaraki	9,500	B-2	LC817210	*15:01/15:01*
EBL111	85	F	HF	Lymph node	Ibaraki	23,006	B-1	LC817211	*10:01/10:01*
EBL112	86	F	HF	Peripheral blood	Ibaraki	6,060	Other	LC817212	*01:01/11:01*
EBL113	87	F	HF	Lymph node	Ibaraki	18,821	B-1	LC817213	*15:01/15:01*
EBL114	89	F	HF	Lymph node	Ibaraki	9,382	A	LC817214	*140:11/15:01*
EBL115	94	F	HF	Solid tumor	Ibaraki	4,140	B-2	LC817215	*15:01/15:01*
EBL116	96	F	HF	Lymph node	Ibaraki	9,992	Other	LC817216	*11:01/11:01*
EBL117	98	F	HF	Lymph node	Ibaraki	3,669	Other	LC817217	*11:01/11:01*
EBL118	98	F	JB	Lymph node	Ibaraki	2,342	B-2	LC817218	*11:01/16:01*
EBL119	100	F	JB	Peripheral blood	Chiba	14,747	A	LC817219	*15:01/15:01*
EBL120	101	F	JB	Lymph node	Ibaraki	7,772	B-2	LC817220	*16:01/16:01*
EBL121	107	F	JB	Lymph node	Ibaraki	6,558	B-2	LC817221	*16:01/16:01*
EBL122	108	F	JB	Peripheral blood	Ibaraki	26,551	B-2	LC817222	*05:04/05:04*
EBL123	115	F	JB	Peripheral blood	Ibaraki	14,998	B-2	LC817223	*07:01/07:01*
EBL124	121	F	HF	Lymph node	Ibaraki	9,921	B-2	LC817224	*16:01/27:03*
EBL125	125	F	JB	Lymph node	Ibaraki	4,921	B-1	LC817225	*15:01/140:11*
EBL126	126	F	HF	Lymph node	Ibaraki	2,886	B-2	LC817226	*16:01/16:01*
EBL127	134	F	JB	Solid tumor	Ibaraki	9,271	B-2	LC817227	*16:01/16:01*
EBL128	140	F	JB	Peripheral blood	Ibaraki	4,124	Other	LC817228	*16:01/16:01*
EBL129	141	F	JB	Peripheral blood	Ibaraki	19,882	Other	LC817229	*15:01/16:01*
EBL130	144	F	JB	Lymph node	Ibaraki	8,291	B-2	LC817230	*16:01/16:01*
EBL131	175	F	JB	Lymph node	Ibaraki	2,819	B-2	LC817231	*05:04/11:01*
EBL132	180	F	JB	Peripheral blood	Ibaraki	8,832	B-2	LC817232	*01:01/05:04*

HF: Holstein-Frisian, JB: Japanese Black
